# 15-Deoxy-***γ***12,14-prostaglandin J2 Reduces Liver Impairment in a Model of ConA-Induced Acute Hepatic Inflammation by Activation of PPAR***γ*** and Reduction in NF-***κ***B Activity

**DOI:** 10.1155/2014/215631

**Published:** 2014-07-10

**Authors:** Kan Chen, Jingjing Li, Junshan Wang, Yujing Xia, Weiqi Dai, Fan Wang, Miao Shen, Ping Cheng, Yan Zhang, Chengfen Wang, Jing Yang, Rong Zhu, Huawei Zhang, Yuanyuan Zheng, Jie Lu, Zhuoyi Fan, Yingqun Zhou, Chuanyong Guo

**Affiliations:** ^1^Department of Gastroenterology, Shanghai Tenth People's Hospital, Tongji University School of Medicine, Shanghai 200072, China; ^2^Tongji University School of Medicine, Shanghai 200092, China

## Abstract

*Objective*. 15-Deoxy-Δ12,14-prostaglandin J2 (15d-PGJ2) reduces inflammation and has been identified as an anti-inflammatory prostaglandin in numerous animal models. In this study, we investigated both effects of 15d-PGJ2 and its protection mechanism in concanavalin A- (ConA-) induced autoimmune hepatitis in mice. *Materials and Methods*. In vivo, Balb/C mice were injected with ConA (25 mg/kg) to induce acute autoimmune hepatitis, and 15d-PGJ2 (10 *μ*g or 25 *μ*g) was administered 1 h before the ConA injection. The histological grade, proinflammatory cytokine levels, and NF-*κ*B and PPAR*γ* activity were determined 6, 12, and 24 h after the ConA injection. In vitro, LO2 cells and RAW264.7 cells were pretreated with 15d-PGJ2 (2 *μ*M) 1 h before the stimulation with ConA (30 *μ*g/mL). The NF-*κ*B and PPAR*γ* activity were determined 30 min after the ConA administration. *Results*. Pretreatment with 15d-PGJ2 reduced the pathological effects of ConA-induced autoimmune hepatitis and significantly reduced the levels of cytokines after injection. 15d-PGJ2 activated PPAR*γ*, blocked the degradation of I*κ*B*α*, and inhibited the translocation of NF-*κ*B into the nucleus. *Conclusion*. These results indicate that 15d-PGJ2 protects against ConA-induced autoimmune hepatitis by reducing proinflammatory cytokines. This reduction in inflammation may correlate with the activation of PPAR*γ* and the reduction in NF-*κ*B activity.

## 1. Introduction

The incidence of hepatitis, or inflammation of the liver, has increased worldwide in the past few decades, threatening human health. Despite the development of several treatments based on research into the mechanism underlying hepatitis, none of them cure the disease. The pathogenesis of hepatitis is multifactorial and includes viral infections, which predominantly trigger hepatitis, autoimmune diseases, metabolic disorders, fatty liver disease, primary biliary cirrhosis, and liver allograft rejection. Many of these cause acute hepatitis, which is characterized by strong innate inflammation, with accompanying cell death, and which may finally lead to liver failure. Activated T lymphocytes play a vital role in this process by infiltrating and destroying the liver parenchyma. Proinflammatory cytokines, such as interleukin 1*β* (IL-1*β*), IL-6, interferon gamma (IFN-*γ*), and tumor necrosis factor alpha (TNF-*α*), also function in the early stages of inflammation [1–3]. Tiegs et al. found that the injection of mice with the T-cell-mitogenic plant lectin concanavalin A (ConA) caused the polyclonal activation of T lymphocytes, mostly CD4^+^ T cells, and induced a liver-specific inflammatory response, including elevated blood levels of IL-2, IL-4, and IFN-*γ* [4, 5]. These features make ConA-induced hepatitis an ideal animal model in which to explore human viral and autoimmune hepatitis.

15-Deoxy-Δ12,14-prostaglandin J2 (15d-PGJ2) was first identified by Fitzpatrick and Wynalda in 1983 [6]. It is derived from prostaglandin (PG) D_2_, a major cyclooxygenase 2 (COX2) product synthesized in a variety of tissues under inflammatory conditions [7]. The *β*-elimination of the hydroxyl group at the C9 position converts PGD_2_ to PGJ_2_, and the latter is subsequently converted into 15d-PGJ2 by albumin-independent dehydration. This process was determined by Shibata et al. using an achiral-phase high-performance liquid chromatography method in 2002 [8]. Studies indicate that both PGD2 and 15d-PGJ2 levels increase in the later phases of COX2 activity, which mediates the resolution of inflammation, whereas early COX2 activity has been associated with a proinflammatory response [9, 10]. After its discovery, the role of 15d-PGJ2 in moderating inflammation has been extensively studied. In the past few decades, 15d-PGJ2 has been shown to reduce inflammation and has been identified as an anti-inflammatory PG in numerous animal models.

The anti-inflammatory effects of 15d-PGJ2 were first described by Gilroy et al. in an animal model of carrageenin-induced inflammation, in which 15d-PGJ2 was substituted for PGE_2_ and played an immunoregulatory role in the resolution of inflammation [9]. Liu et al. also found that 15d-PGJ2 reduced the general activity of both RAW264.7 and J774A.1 macrophages [11]. Moreover, 15d-PGJ2 not only reduces the production of cytokines secreted by monocytes but also downregulates the recruitment of bone-marrow monocytes during chronic liver inflammation [12, 13] and reduces the phagocytic activities of bone-marrow macrophages in vitro [13]. Alves et al. used nanocapsules loaded with 15d-PGJ2 to reduce neutrophil migration, as well as IL-1*β*, TNF-*α*, and IL-12p70 production, during inflammation, which proved an effective strategy for attenuating inflammation [14]. The function of 15d-PGJ2 against microbial infections has also been extensively studied. Dugo et al. [16] found that treatment with 15d-PGJ2 helped to reduce vascular injury and attenuated hypotension; reduced cytokine production; alleviated neutrophil infiltration into the lung, colon, and liver, thus ameliorating the dysfunction of these organs; and resulted in increased survival after both Gram-positive and Gram-negative bacterial challenge. This makes 15d-PGJ2 a potential therapeutic agent against sepsis and septic shock [15, 16]. Intriguingly, 15d-PGJ2 can help reduce the morbidity and mortality of mice infected with the influenza virus [17]. Investigation of the mechanism of this anti-inflammatory PG began with a study of its influence on the activity of NF-*κ*B, a protein with various transcriptional regulatory functions, which is critical in the inflammatory responses. Rossi et al. reported that 15d-PGJ2 inhibits NF-*κ*B activation by covalently binding to and consequently inhibiting I*κ*B kinase (IKK), which phosphorylates I*κ*B*α*, a major inhibitor of NF-*κ*B activation, thus inducing the proteasomal degradation of I*κ*B*α* [18]. Shiraki et al. also found that 15d-PGJ2 can act as a natural ligand of peroxisome proliferator-activated receptor gamma (PPAR*γ*) by binding via a Michael addition reaction [19]. PPAR*γ* has recently been shown to have anti-inflammatory activity by reducing the DNA-binding activity of NF-*κ*B and suppressing its translocation into the nucleus, which attenuates cytokine production and neutrophil infiltration and finally reduces tissue injury [20, 21]. Since the effects and mechanism of 15d-PGJ2 in mediating inflammation have been confirmed, this PG has been investigated as an effective therapeutic agent for a variety of inflammatory diseases, including rheumatoid arthritis, atherosclerosis, myocardial infarction, cerebral injury, acute pancreatitis, and gastrointestinal injury [22–26]. Despite our knowledge and extensive exploration of the roles of 15d-PGJ2 in numerous inflammatory diseases, it is still unclear whether it influences autoimmune liver diseases, and the probable mechanisms also require investigation.

In this study, we hypothesize that 15d-PGJ2 reduces the impairment associated with ConA-induced acute hepatic inflammation by reducing the production of inflammatory mediators, thus attenuating liver injury. The mechanism may be attributable to its effect on the NF-*κ*B pathway.

## 2. Materials and Methods

### 2.1. Reagents

ConA and 15d-PGJ2 were purchased from Sigma Aldrich (St. Louis, MO, USA). The antibodies used in this study include those directed against NF-*κ*B (Abcam, USA), PPAR*γ* (Cell Signaling Technology, USA), and I*κ*B*α* (Cell Signaling Technology, USA). The alanine aminotransferase (ALT) and aspartate aminotransferase (AST) microplate test kits were purchased from Nanjing Jiancheng Bioengineering Institute (Jiancheng Biotech, China).

### 2.2. Animals

Male Balb/c mice (8 weeks old, 23 ± 2 g) were purchased from Shanghai SLAC Laboratory Animal Co. Ltd. (Shanghai, China). The mice were housed in a clean room maintained at 24 ± 2°C under a 12 h : 12 h light : dark cycle, with free access to food and water. All animal experiments were approved by the Animal Care and Use Committee of Shanghai Tongji University.

### 2.3. Drug Administration

Ninety-six mice were used in this study, divided randomly into four groups:

1. normal control (injected with vehicle or 10 *μ*g 15d-PGJ2 or 25 *μ*g 15d-PGJ2);
2. ConA injected;
3. ConA + 10 *μ*g 15d-PGJ2;
4. ConA + 25 *μ*g 15d-PGJ2.

15d-PGJ2 (dissolved in methyl acetate) was diluted with phosphate-buffered saline (PBS) and injected intraperitoneally 1 h before the injection of ConA. ConA was dissolved in pyrogen-free normal saline solution at a concentration of 5 mg/mL and injected via the tail vein at a dose of 25 mg/kg bodyweight to induce acute hepatic injury.

### 2.4. Cell Lines and Culture

The LO2 cells and RAW264.7 cells were purchased from Chinese Academy of Science Committee Type Culture Collection Cell Bank. Both cell lines were cultured in high glucose Dulbecco's modified Eagle's medium (Gibco, USA) supplemented with 10% fetal bovine serum (HyClone, South America) and 1% penicillin-streptomycin (Gibco, USA) in a humidified incubator at 37°C in 5% CO_2_.

Each kind of cells was divided into four groups:

1. treated with PBS only as vehicle;
2. treated with 15d-PGJ2 diluted in PBS at a concentration of 2 *μ*M;
3. treated with ConA dissolved in PBS solution at a concentration of 30 *μ*g/mL;
4. 15d-PGJ2 treated 1 h before the stimulation with ConA.

### 2.5. Biochemical Analysis

Six randomly selected mice from each group were killed at the each time point: 6 h, 12 h, and 24 h after ConA injection. We collected their liver tissues (stored at −80°C) and orbital blood (stored at 4°C).

#### 2.5.1. Serum Aminotransferase Assessment

After storage at 4°C for 4-5 h, the sera were separated from the orbital blood by centrifugation at 2000 rcf for 10 min at room temperature. As indices of hepatocellular injury, the serum levels of ALT and AST were measured with ALT and AST microplate test kits, according to the manufacturer's instructions.

#### 2.5.2. Serum Cytokine Assays

Enzyme-linked immunosorbent assay (ELISA) kits (R&D Systems, USA) were used according the manufacturers' protocols to measure serum proinflammatory cytokines TNF-*α*, IL-2, IL-6, and IL-12.

### 2.6. Histopathology

The middle portion of the left lobe of the liver of each mouse was excised and sectioned and then perfused in 4% paraformaldehyde for at least 24 h. After fixation, the tissues were embedded in paraffin, and 5 *μ*m thick sections were then stained with hematoxylin and eosin (H&E) to observe the tissue damage by light microscopy.

### 2.7. Immunohistochemistry

After heating in a baking oven at 60°C for 20 min, the prepared paraffin-embedded sections were dewaxed in dimethylbenzene and rehydrated through a graded series of alcohol. To recover the antigens, the paraffin-embedded slices were immersed in citrate buffer and incubated in a 95°C water bath for 20 min. The sections were then covered in 3% hydrogen peroxide for 20 min at 37°C to block endogenous peroxidase activity. Nonspecific binding sites were blocked with 5% bovine serum albumin at 37°C for 20 min and then incubated at room temperature for 10 min. The liver sections were incubated overnight with rabbit anti-mouse PPAR*γ* antibody (diluted 1 : 100) and rabbit anti-mouse NF-*κ*B/p65 antibody (diluted 1 : 100). On day 2, the slices were incubated with goat anti-rabbit secondary antibody (Epitomics, CA) for 30 min at room temperature. The analysis of antibody binding was performed with a diaminobenzidine (DAB) kit, and hematoxylin was used as the counterstain. The slices were dehydrated through graded ethanol and xylene and mounted with Entellan. The slides were then observed by light microscopy.

### 2.8. Western Blotting Analysis

After they were ground in liquid nitrogen, the liver tissues were rapidly lysed with RIPA lysis buffer containing PMSF, aprotinin, sodium orthovanadate, and sodium fluoride (Sigma, USA). Nuclear extract kit (Pierce, USA) was used to lyse cells and collect the nuclear proteins following the manufacturer's protocol 30 min after the ConA administration. The protein concentration was then determined with the bicinchoninic acid (BCA) method. Equivalent amounts of total protein (30–120 *μ*g) were boiled with 5 × SDS-PAGE sample loading buffer. The treated samples were analyzed with SDS-PAGE according to standard protocols. Nonspecific binding was blocked with 5% nonfat milk (dissolved in PBS) for 2 h and the blots were then incubated overnight at 4°C with rabbit antibodies directed against mouse *β*-actin (1 : 1000), mouse PPAR*γ* (1 : 400), mouse NF-*κ*B/p65 (1 : 400), or mouse I*κ*B*α* (1 : 200) diluted in 5% milk. *β*-Actin was used as the internal reference for cytoplasmic proteins and Lamin-A for nuclear proteins. All membranes were washed three times with PBS containing 0.1% Tween 20 (PBST) and then incubated with a secondary goat anti-mouse or anti-rabbit antibody (1 : 2000) dissolved in PBST, at 37°C for 45 min. Finally, the membranes were washed three times with PBST for 5 min each and the proteins were detected using the Odyssey two-color infrared laser imaging system (fluorescence detection).

### 2.9. Immunofluorescence

The cells were first washed three times with PBS solution for 1 min and then fixed with 4% paraformaldehyde for 10 min. After being washed with PBS solution three times for 3 min each, the cells were permeabilized in 0.3% Triton X-100 (Sigma, USA) for 10 min and washed with PBS again. The cells were blocked with 5% bovine serum albumin (BSA) in PBS for 30 min and incubated with rabbit anti-mouse PPAR*γ* antibody (diluted 1 : 100) and rabbit anti-mouse NF-*κ*B/p65 antibody (diluted 1 : 100) overnight at 4°C. In the following morning, the slides were washed with PBS solution and incubated with appropriate fluorescein isothiocyanate-conjugated secondary antibody for 1 h. The cells were subsequently washed and incubated with 2-(4-amidinophenyl)-6-indolecarbamidine dihydrochloride (DAPI) (Life Technologies, USA) 3 min for nuclear staining. The slides were finally visualized with LSM710 Carl Zeiss confocal microscope (Carl Zeiss AG, Germany).

### 2.10. Real-Time Reverse Transcriptase-Polymerase Chain Reaction (qRT-PCR)

The liver tissue transcripts were detected and analyzed with qRT-PCR. Total RNA was extracted from frozen liver tissues with TRIzol reagent (Tiangen Biotech, China), as instructed by the manufacturer. SYBR Green Quantitative RT-PCR was performed with the 7900HT Fast Real-Time PCR system (ABI, CA, USA) to determine the expression of the target genes, according to the instructions for SYBR Premix EX Taq (TaKaRa Biotechnology, China). The primer sequences are shown in Table 1.

### 2.11. Statistical Analysis

All results are expressed as means ± SD. The qPCR and ELISA data were analyzed with one-way analysis of variance (ANOVA). The serum levels of ALT and AST, the ratio of cellular necrotic or edematous areas in histopathology and the positive cell rate in immunohistochemistry were analyzed with Student's *t*-test. In all comparisons, *P* < 0.05 was considered statistically significant. All statistical analyses were performed with SPSS 17.0 for Windows.

## 3. Results

### 3.1. 15d-PGJ2 Pretreatment Reduced ConA-Induced Liver Injury in Mice

**Figure 1:**
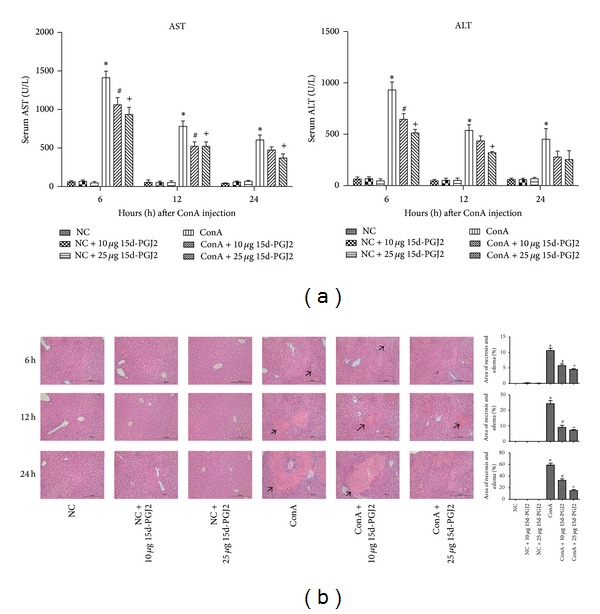
15d-PGJ2 pretreatment group attenuate ConA-induced acute hepatitis. (a) The index of plasma ALT and AST levels at 6 h, 12 h, and 24 h after ConA injection in mice and effects of both low (10 *μ*g) and high (25 *μ*g) dose 15d-PGJ2 pretreatment groups at the same time. Data are expressed as mean ± SD (*n* = 6, *P < 0.05 for NC versus ConA, ^#^ *P* < 0.05 for ConA versus ConA + 10 *μ*g 15d-PGJ2, and ^+^ *P* < 0.05 for ConA versus ConA + 25 *μ*g 15d-PGJ2). (b) Hematoxylin and eosin staining of liver sections (NC or NC + 10 *μ*g 15d-PGJ2 or NC + 25 *μ*g 15d-PGJ2 or ConA or ConA + 10 *μ*g 15d-PGJ2 or ConA + 25 *μ*g 15d-PGJ2). The cellular necrotic or edematous areas were analyzed with Image-Pro Plus 6.0, indicating there existed statistical significance among different groups (*n* = 6, *P < 0.05 for NC versus ConA, ^#^ *P* < 0.05 for ConA versus ConA + 10 *μ*g 15d-PGJ2, ^+^ *P* < 0.05 for ConA versus ConA + 25 *μ*g 15d-PGJ2, black bar for 200 *μ*M, wide arrow for necrotic area, and narrow arrow for edematous area).

Figure 1(a) shows that, after the ConA injection, the serum levels of ALT and AST increased statistically significantly at each designated time point compared with the normal control group. Meanwhile, the quantitative value of the ALT and AST in pretreatment of both low and high dose of 15d-PGJ2 group decayed in the same point. In the plasma ALT, the statistical distinctions appeared in two pretreatment groups at 6 h while only the high dose 15d-PGJ2 group exhibited statistical difference at 12 h. However, no pretreatment groups had a statistical reduction in 24 h. With respect to plasma AST, two pretreatment groups had statistical effects at 6 and 12 h when compared to ConA model group. But the obvious protective effect was only exhibited in the high dose of 15d-PGJ2 group at 24 h. The histopathological analysis showed marked necrosis in the ConA-induced group, as shown in Figure 1(b). The severity of necrosis increased with time, with the most massive area of nuclear fragmentation being apparent after 24 h. Although cell death was observed in the 15d-PGJ2-pretreated groups, it was clearly less than that in the ConA-treated groups. These results demonstrate the direct positive effects of pretreatment with 15d-PGJ2 on ConA-induced liver injury. In addition, injection alone of 15d-PGJ2 in both low and high doses will not cause the elevation of ALT and AST level in serum compared with NC group (Figure 1(a)). Also, neither cellular necrotic nor edematous areas are observed (Figure 1(b)), indicating that 15d-PGJ2 of these two doses has no side effects on the mice in normal group.

### 3.2. Pretreatment with 15d-PGJ2 Reduced the Production of IL-2, IL-6, IL-12, and TNF-*α* in ConA-Induced Hepatitis

**Figure 2:**
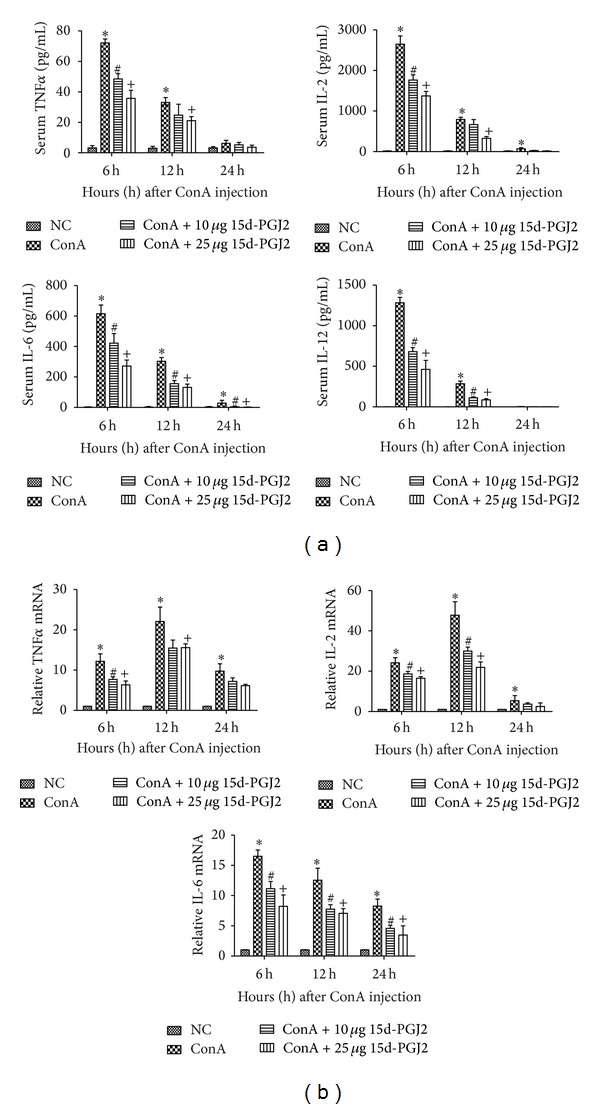
Pretreatment with 15d-PGJ2 reduced the production of IL-2, IL-6, IL-12, and TNF-*α* in ConA-induced hepatitis. (a) The index of plasma IL-2, IL-6, IL-12, and TNF-*α* levels at 6 h, 12 h, and 24 h after ConA injection in mice and effects of both low (10 *μ*g) and high (25 *μ*g) dose 15d-PGJ2 pretreatment groups at the same time. Data are expressed as mean ± SD (*n* = 6, *P < 0.05 for NC versus ConA, ^#^ *P* < 0.05 for ConA versus ConA + 10 *μ*g 15d-PGJ2, and ^+^ *P* < 0.05 for ConA versus ConA + 25 *μ*g 15d-PGJ2). (b) The mRNA expression of IL-2, IL-6, IL-12, and TNF-*α* in NC, ConA, ConA + 10 *μ*g 15d-PGJ2, and ConA + 25 *μ*g 15d-PGJ2 group was evaluated by Real-Time PCR (*n* = 6, *P < 0.05 for NC versus ConA, ^#^ *P* < 0.05 for ConA versus ConA + 10 *μ*g 15d-PGJ2, and ^+^ *P* < 0.05 for ConA versus ConA + 25 *μ*g 15d-PGJ2).

The ELISA results indicated that all the measured proinflammatory cytokines increased significantly in the mice plasma at 6 and 12 h after ConA injection. The plasma IL-2 and IL-6 levels still statistically increased at 24 h compared with the normal control group, shown in Figure 2(a). We used qRT-PCR to analyze the changes of these cytokines in tissues of these experimental mice. The transcriptional levels of IL-2, IL-6, and TNF-*α* were similarly upregulated in all the tested time points, shown in Figure 2(b). In the index of IL-2 and TNF-*α*, the peak of mRNA level appeared at 12 h, while the peak of plasma appeared at 6 h. The tendency of increasing in IL-6 stayed the same and peaked at 6 h. The high dose of 15d-PGJ2 pretreatment group still exhibited statistical reduction in both plasma and mRNA levels of IL-2, IL-6, and TNF-*α* at 6 and 12 h compared with ConA group. Likewise, the high dose of 15d-PGJ2 group affected IL-6 in both plasma and mRNA levels at 24 h. The low dose of 15d-PGJ2 pretreatment group reduced IL-2, IL-12, and TNF-*α* in plasma levels mainly at 6 h. Its effect on IL-6 is consistent with the high dose group.

### 3.3. 15d-PGJ2 Activated PPAR*γ*, Blocked I*κ*B*α* Degradation, and Reduced the NF-*κ*B Activation in ConA-Induced Hepatitis

**Figure 3:**
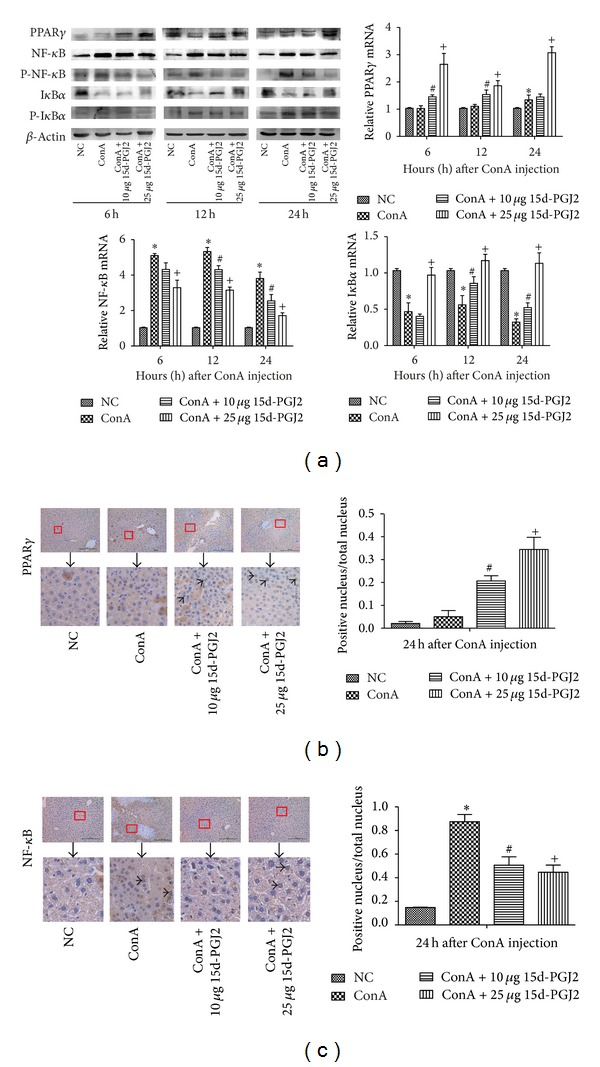
15d-PGJ2 activated PPAR*γ*, blocked I*κ*B*α* degradation, and reduced the NF-*κ*B activation in ConA-induced hepatitis. (a) The western blot and qRT-PCR analysis of PPAR*γ*, I*κ*B*α*, and NF-*κ*B at 6 h, 12 h, and 24 h after ConA injection in mice and effects of both low (10 *μ*g) and high (25 *μ*g) dose 15d-PGJ2 pretreatment groups at the same time. The phosphorylation status of NF-*κ*B and I*κ*B*α* (P-NF-*κ*B and P-I*κ*B*α*) were also detected by using western blot. The results were analyzed using Quantity One (*n* = 3, *P < 0.05 for NC versus ConA, ^#^ *P* < 0.05 for ConA versus ConA + 10 *μ*g 15d-PGJ2, and ^+^ *P* < 0.05 for ConA versus ConA + 25 *μ*g 15d-PGJ2). (b) Immunohistochemistry used to detect the expression level of PPAR*γ* at 24 h in all four groups. The result was analyzed using Image-Pro Plus 6.0 (*n* = 3, ^#^ *P* < 0.05 for ConA versus ConA + 10 *μ*g 15d-PGJ2, ^+^ *P* < 0.05 for ConA versus ConA + 25 *μ*g 15d-PGJ2, and black bar for 200 *μ*M). (c) The different expression of NF-*κ*B evaluated by immunohistochemistry. Image-Pro Plus 6.0 was used to analyze whether there exists statistical significance among different groups (*n* = 3, *P < 0.05 for NC versus ConA, ^#^ *P* < 0.05 for ConA versus ConA + 10 *μ*g 15d-PGJ2, ^+^ *P* < 0.05 for ConA versus ConA + 25 *μ*g 15d-PGJ2, black bar for 200 *μ*M, and small black arrow for positive cells).

We investigated the levels of PPAR*γ*, I*κ*B*α*, and NF-*κ*B by using western blotting and qRT-PCR. As Figure 3(a) shows, the protein and mRNA levels of I*κ*B*α* were both reduced in the ConA-treated group compared with normal control group at the same time points. While both the low and high doses of 15d-PGJ2-pretreated groups ameliorated the degradation of I*κ*B*α* in protein level and increased the mRNA expression level at 12 and 24 h compared with the ConA-treated group, the high dose of 15d-PGJ2-pretreated group upregulated both the protein and mRNA levels of PPAR*γ* at all designed time points, in contrast with normal control and ConA-treated groups. The changes were statistically significant. To further investigate whether there were changes in the phosphorylated I*κ*B*α* and NF-*κ*B (P-I*κ*B*α* and P-NF-*κ*B), known as the activated condition, we subsequently detected the expression of P-I*κ*B*α* and P-NF-*κ*B by western blotting. 15d-PGJ2-pretreated group had a lower level of phosphorylated I*κ*B*α* compared with ConA-treated group, indicating less degradation of I*κ*B*α*. Besides, 15d-PGJ2-pretreated groups not only reduced the protein and mRNA expressions at 12 and 24 h in comparison with ConA-treated group but also decreased the phosphorylation level of NF-*κ*B and thus prevented its translocation into the nucleus, shown in Figures 3(a) and 3(c). With respect to PPAR*γ*, the immunohistochemistry graphs display an obvious augment in PPAR*γ* between the ConA-treated group and the 15d-PGJ2-pretreated group (Figure 3(b)).

### 3.4. 15d-PGJ2 Upregulated PPAR*γ* Expression in Liver Cells (LO2) Nuclei and Suppressed the NF-*κ*B Activation in Macrophages (RAW264.7)

**Figure 4:**
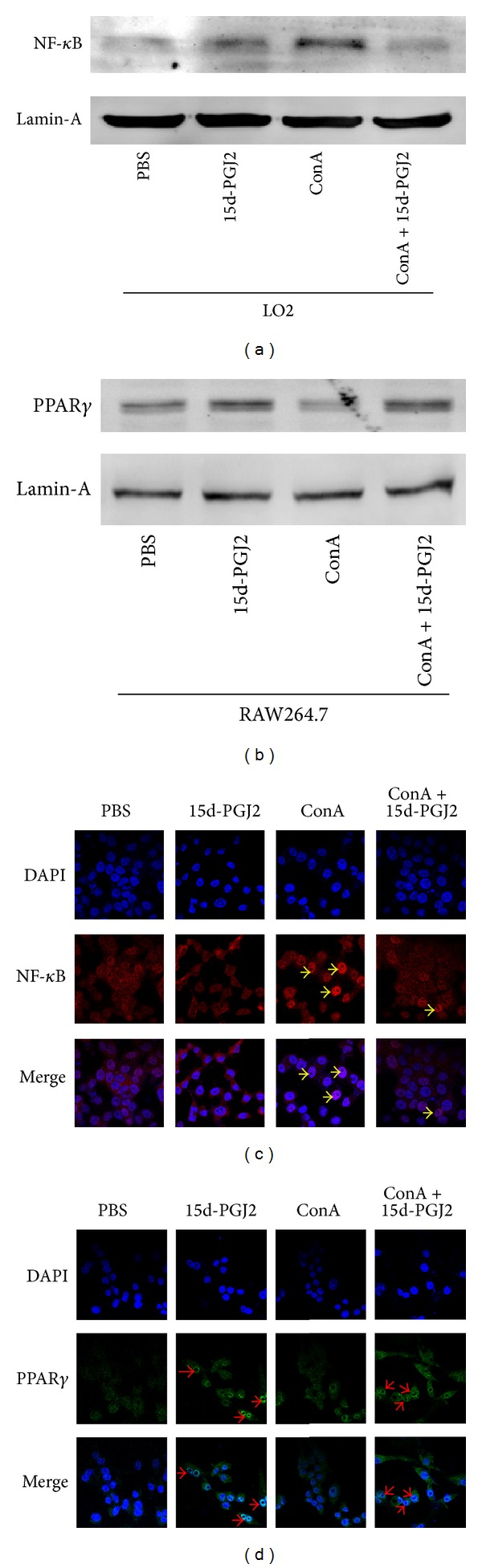
15d-PGJ2 upregulated PPAR*γ* expression in liver cells (LO2) nuclei and suppressed the NF-*κ*B activation in macrophages (RAW264.7). (a) The levels of NF-*κ*B in the nuclei of LO2 cells (treated with PBS or 2 *μ*M 15d-PGJ2 or 30 *μ*g/mL ConA or 15d-PGJ2 + ConA) were detected by western blot. (b) The western blot was used to analyze the levels of PPAR*γ* in RAW264.7 cell nucleus (the same treatment and grouping as LO2 cells). (c) The different expression of NF-*κ*B in LO2 cellular localization were evaluated by immunofluorescence (magnification is 630x, yellow arrow for positive cells). (d) The expression of PPAR*γ* in RAW264.7 cells was detected by immunofluorescence (magnification is 630x, red arrow for positive cells).

To prove the direct effect of 15d-PGJ2 in vitro, we also conducted the cytology experiment. LO2 and RAW264.7 cells were both treated with or without 15d-PGJ2 for 1 h and subsequently incubated with ConA. The western blot and immunofluorescence were used to detect the expression of NF-*κ*B and PPAR*γ* in cell nucleus. 15d-PGJ2 obviously inhibited the activation and translocation of NF-*κ*B into liver cells nuclei induced by ConA and reduced the expression of NF-*κ*B protein in nuclear, shown in Figures 4(a) and 4(c). Besides, 15d-PGJ2 could increase the activation of PPAR*γ* and its nuclear translocation in macrophages with or without ConA, as shown in Figures 4(b) and 4(d).

## 4. Discussion

In this study, we used ConA to induce acute liver injury to create a model of liver injury in which to characterize CD4^+^ T-cell activation, subsequent inflammation, the kinds of cell death, and possibly ultimate liver failure. Our results show that ConA-induced liver injury was ameliorated in 15d-PGJ2-pretreated mice, and this was accompanied by a reduction in the expression of proinflammatory cytokines and NF-*κ*B activation.

The serum levels of ALT and AST were markedly elevated in the ConA-treated group compared with those in the normal group. The serum levels of ALT and AST are elevated after hepatocyte death and the release of the transaminases into the blood. Hepatocyte death caused by inflammation was clearly observed with H&E staining. The region of punctiform necrosis at 6 h after ConA injection had increased massively at the 24 h time point, together with an increase in cellular edema. However, the serum ALT and AST levels were reduced in the 15d-PGJ2-pretreated groups relative to those in the ConA-treated groups at most time point. The areas of cell death in the 15d-PGJ2-pretreated group were clearly reduced, as shown in Figure 1(b). Therefore, 15d-PGJ2 pretreatment clearly attenuated the acute liver injury induced by ConA. The modulatory function of 15d-PGJ2 in inflammation was reflected in the downregulation of proinflammatory cytokines, such as IL-2, IL-6, and TNF-*α*. The effect was marked at both the protein and mRNA levels, as shown in Figure 2. The anti-inflammatory effects of 15d-PGJ2 were explicit and consistent with other studies of a variety of inflammatory diseases [27–30]. IL-12 is a macrophage-derived proinflammatory cytokine that plays a crucial role in T-cell proliferation and CD4^+^ T-cell differentiation. It has also been reported that inhibiting the IL-12 signaling pathway attenuates CD4^+^ T-cell-mediated autoimmune diseases, including experimental allergic encephalomyelitis [31, 32]. To examine this feature in our experimental model, we also measured the IL-12 levels. Our results indicated that the plasma IL-12 level was clearly elevated at all designed time points, peaking at 6 h. The 15d-PGJ2-pretreated group showed specifically reduced IL-12 production, as was reported by Alves et al. [14] and Natarajan and Bright [32]. 

Unlike other classes of eicosanoids, 15d-PGJ2 has an *α*,*β*-unsaturated ketone located in a cyclopentenone ring, which makes 15d-PGJ2 more electrophilic and reactive, covalently modifying critical proteins in multiple pathways. The anti-inflammatory effects of 15d-PGJ2 are attributed to its covalent binding to members of the NF-*κ*B and PPAR*γ* pathways [18, 33]. As mentioned above, 15d-PGJ2 can block NF-*κ*B activation, inhibiting the phosphorylation of I*κ*B*α* and its subsequent degradation. Our data show that the I*κ*B*α* levels in all the mice in the high dose of 15d-PGJ2-pretreated group were markedly higher than those in the ConA-injected group at all the time points tested, as shown in Figure 3(a). This prevented the translocation of NF-*κ*B into the nucleus. Immunohistochemistry identified the different localization of NF-*κ*B in the different groups. As shown in Figure 3(b). NF-*κ*B was predominantly expressed and located in the hepatocyte nuclei in the ConA-induced group, whereas the 15d-PGJ2-treated group showed a clear reduction in nuclear-localized NF-*κ*B. Our findings are corroborated by numerous studies [29, 34–36]. Another target covalently bound by 15d-PGJ2 is PPAR*γ*. As shown in Figure 3(a), PPAR*γ* was elevated at both the mRNA and protein levels in the 15d-PGJ2-treated group compared with the normal and ConA-induced-hepatitis groups. The immunohistochemistry results also demonstrated the strong expression and localization of PPAR*γ* in the hepatic cells of the 15d-PGJ2-treated group, shown in Figure 3(c). All these data confirm that 15d-PGJ2 activates PPAR*γ* in ConA-induced hepatitis. Intriguingly, PPAR*γ* exerts its anti-inflammatory response via a number of mechanisms, including the NF-*κ*B pathway and the nuclear factor of activated T-cells (NF-AT) pathway [37]. A study by Li et al. indicated that PPAR*γ* reduces the DNA-binding activity of NF-*κ*Bp65 and subsequently inhibits the expression of genes downstream in the inflammatory response and ameliorates sepsis-induced liver injury. An ELISA-based Trans-AM Transcription Factor Assay was used in that study [21]. Using an electrophoresis mobility shift assay, Ogawa et al. also demonstrated the suppression of the DNA-binding activity of NF-*κ*B by PPAR*γ* activation in ConA-induced hepatitis [20]. This conclusion is confirmed by our immunohistochemistry results. 15d-PGJ2 was used to activate PPAR*γ* in neither of these experiments, suggesting that the activation of PPAR*γ* also influences the DNA-binding activity of NF-*κ*B independent of 15d-PGJ2. In summary, 15d-PGJ2 reduces the release of proinflammatory cytokines in ConA-induced acute liver injury by blocking the activation of NF-*κ*B, and the activation of PPAR*γ* by 15d-PGJ2 participates in this process.

Here, we have shown for the first time that 15d-PGJ2 reduces liver impairment in a model of ConA-induced acute hepatic inflammation. The role of 15d-PGJ2 as an anti-inflammatory PG is consistent with the results of previous researches. The major mechanism by which 15d-PGJ2 mediates the inflammatory response is its inhibition of NF-*κ*B activation, and the PPAR*γ* pathway is also involved. The effect of 15d-PGJ2 on regulation of NF-*κ*B and PPAR*γ* activation in vitro has also been investigated. This study suggests that 15d-PGJ2 is a potential therapeutic agent on autoimmune hepatitis.

## Figures and Tables

**Table 1:** 

Gene		Primer sequence (5′ → 3′)
PPAR*γ*	Forward	GGAAGACCACTCGCATTCCTT
	Reverse	GTAATCAGCAACCATTGGGTCA

IL-6	Forward	CTGCAAGAGACTTCCATCCAG
	Reverse	CCCCACCGAACTCAAAGAAGG

IL-2	Forward	TGAGCAGGATGGAGAATTACAGG
	Reverse	GTCCAAGTTCATCTTCTAGGCAC

I*κ*B*α*	Forward	GCCCCGCACAGCCATGTTTC
	Reverse	AGCGGACAGGCGAGGAGAGC

NF-*κ*b/p65	Forward	ATGGCAGACGATGATCCCTAC
	Reverse	CGGATCGAAATCCCCTCTGTT

*β*-actin	Forward	GGCTGTATTCCCCTCCATCG
	Reverse	CCAGTTGGTAACAATGCCATGT

TNF-*α*	Forward	CAGGCGGTGCCTATGTCTC
	Reverse	CGATCACCCCGAAGTTCAGTAG
